# Ethical Relativism Mediates the Association Between Openness to Experience and Negative Creativity

**DOI:** 10.3390/bs16071165

**Published:** 2026-07-10

**Authors:** Marco Giancola, Laura Piccardi, Simonetta D’Amico, Raffaella Nori, Paola Guariglia, Emanuele Cardella, Massimiliano Palmiero

**Affiliations:** 1 Department of Biotechnological and Applied Clinical Sciences, University of L’Aquila, 67100 L’Aquila, Italy; marco.giancola@univaq.it (M.G.); simonetta.damico@univaq.it (S.D.); 2 Department of Psychology, “Sapienza” University of Rome, 00185 Rome, Italy; laura.piccardi@uniroma1.it; 3 San Raffaele Cassino Hospital, 03043 Cassino, Italy; 4 Department of Psychology, University of Bologna, 40127 Bologna, Italy; raffaella.nori@unibo.it; 5 Department of Human and Social Sciences, Mercatorum University, 00185 Rome, Italy; paola.guariglia@unimercatorum.it; 6 Department of Human and Social Sciences, University of Enna “Kore”, 94100 Enna, Italy; emanuele.cardella@unikorestudent.it; 7 Department of Communication Sciences, University of Teramo, 64100 Teramo, Italy

**Keywords:** personality, creative production, ethics, relativism, mediation, moral reasoning

## Abstract

The present study investigated whether relativism accounted for the association between openness to experience and two distinct forms of creativity: positive creativity, defined as original and beneficial ideas, and negative creativity, defined as original ideas that may unintentionally result in harm. A sample of 200 participants (M_age_ = 21.79 years, SD_age_ = 2.64 years; 146 females) completed the Openness to Experience subscale of the HEXACO Personality Inventory–Revised (HEXACO-PI-R) and the Relativism subscale of the Ethics Position Questionnaire (EPQ-5). Positive and negative creativity were evaluated by two independent judges using a modified Cartoon Caption Task, in which participants generated captions for five New Yorker-style cartoons. Results indicated that relativism accounted for only the association between openness to experience and negative creativity. Specifically, individuals higher in openness to experience tend to produce more ethically ambiguous creative captions when they also endorse a flexible, context-dependent moral perspective. By contrast, positive creativity was more directly associated with dispositional curiosity and imagination. These findings contribute to research on the adaptive and maladaptive sides of creativity by suggesting that moral reasoning may be associated with the ethical valence of creative ideation. The study also highlights the importance of integrating personality and ethical perspectives into creativity research and provides implications for educational programmes aimed at fostering responsible, socially constructive innovation. Limitations and future research directions are discussed.

## 1. Introduction

Creativity is the ability to produce ideas, objects, or solutions that are both novel and appropriate within a given social or cultural context (Brandt, 2021; Giancola et al., 2022; Runco, 2025). As a higher-order form of human cognition, creativity has traditionally been associated with constructive and socially valued outcomes, including artistic expression, scientific discovery, adaptive problem solving, innovation, sustainability, health, and well-being (Aydeniz & Stone, 2023; Giancola et al., 2024a; Jean-Berluche, 2024; Walia, 2019; Sannino et al., 2024). This constructive view is captured by the concept of positive creativity, which refers to original and useful ideas, products, or behaviours that generate beneficial, prosocial, or socially constructive consequences (Beghetto & Anderson, 2022; Cropley et al., 2008; Cropley, 2010; Sternberg, 2022).

However, creativity is not inherently benevolent. Research has challenged this implicit “benevolence bias”, showing that creativity can also facilitate harmful, exploitative, or morally ambiguous goals (Baas et al., 2019; Logan et al., 2023; Perchtold-Stefan et al., 2021). This line of research has led to a broader conceptualisation of the dark side of creativity, namely the use of original means to achieve harmful outcomes (McLaren, 1993). Within this framework, negative creativity can be understood as the production of novel and effective ideas, products, or behaviours that generate harm without malicious intent (Kapoor & Kaufman, 2022).

Within the field of individual differences, the relationship between personality and positive creativity has been extensively examined (Feist, 1998; Kaufman et al., 2016). Among personality traits, openness to experience—characterised by intellectual curiosity, imagination, cognitive flexibility, and aesthetic sensitivity—has emerged as one of the most consistent dispositional predictors of creativity (Wolfradt & Pretz, 2001). Longitudinal research further suggests that openness to experience during adolescence predicts higher levels of creative engagement and creative accomplishment across the lifespan (Asquith et al., 2024; Soldz & Vaillant, 1999). Importantly, openness to experience does not appear to be associated exclusively with constructive creative outcomes. Individuals high in this trait, particularly those characterised by vivid imagination and intellectual curiosity, may also be more likely to generate negative creative ideas, even when the harmful consequences of such ideas are unintended (Kapoor & Khan, 2020).

Despite this evidence, the ethical processes involved in the association between openness to experience and creativity remain underexplored. In particular, little attention has been paid to whether ethical positions may help explain why openness to experience is associated with both positive and negative creativity. Ethics involves cognitive, affective, and socio-emotional processes through which individuals evaluate actions, distinguish right from wrong, and regulate behaviour in accordance with internalised moral standards and social norms (Caravita et al., 2018; Cohen et al., 2014; D. Forsyth, 2020). According to Ethics Position Theory (D. R. Forsyth, 1980; D. Forsyth, 2020), individuals differ in their ethical ideologies along two orthogonal dimensions: idealism and relativism. Idealism holds that morally appropriate actions should avoid harm and promote desirable consequences for others, whereas relativism rejects universal moral rules and evaluates ethical issues according to contextual, cultural, and situational contingencies (D. R. Forsyth, 1980; D. Forsyth, 2020; O’Boyle & Forsyth, 2021).

Research has suggested a positive association between openness to experience and relativism (Khan et al., 2016; Mudrack & Mason, 2020; Sarkissian, 2016). This association is theoretically reasonable as individuals high in openness to experience are typically more willing to explore multiple perspectives, tolerate ambiguity, and question conventional assumptions (A. P. Christensen et al., 2019; Feist, 1998; Kaufman et al., 2016; Hiel & Mervielde, 2004). Rather than relying exclusively on fixed moral rules, they may be more inclined toward nuanced, context-sensitive reasoning. Such ethical flexibility may be relevant to creative ideation because creativity often requires the capacity to move beyond established schemas, challenge dominant assumptions, and generate responses that are not immediately constrained by conventional expectations.

Indirect evidence further supports the potential link between relativism and creativity. Creative cognition has been associated with deception, dishonesty, rule-breaking, and ethically questionable behaviours, suggesting that originality may sometimes be accompanied by reduced adherence to conventional moral constraints (Gino & Ariely, 2012; Storme et al., 2021). Moreover, meta-analytic findings indicate a small but positive association between Machiavellianism and creativity, suggesting that creative potential may coexist with strategic, self-serving, and norm-violating tendencies (Lebuda et al., 2021). These findings indicate that creative ideation and ethical flexibility may intersect when individuals generate unconventional solutions to ambiguous or socially complex situations.

Taken together, these findings suggest that relativism may constitute a meaningful variable in the association between openness to experience and both positive and negative creativity. Importantly, this does not imply that relativism directly determines whether creativity will be beneficial or harmful. In this regard, relativism may be relevant across both domains, as both positive and negative creativity require some degree of departure from fixed, conventional, or normatively prescribed ways of thinking. By reducing reliance on universal moral rules and increasing sensitivity to contextual contingencies, a relativistic orientation may allow individuals high in openness to evaluate ideas less in terms of absolute moral prescriptions and more in terms of situational meaning, pragmatic consequences, and contextual appropriateness. This normative flexibility may support positive creativity when unconventional ideas are directed toward constructive, prosocial, humorous, or adaptive outcomes. In addition, it may support negative creativity when unconventional ideas are directed toward transgressive, hostile, grotesque, or otherwise harmful outcomes, particularly when the interpersonal or motivational context favours such aims (Lee & Dow, 2011).

**Figure 1:**
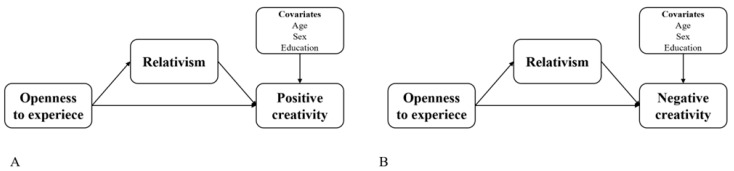
Summary of the hypothesised mediating role of relativism in the association between openness to experience and both positive (**A**) and negative creativity (**B**).

Accordingly, the present study investigated whether relativism accounts for the indirect association between openness to experience and both positive and negative creativity, while controlling for age, sex, and education. These covariates were included as creativity is shaped by developmental and socio-educational factors. Age has been associated with variation in divergent thinking and creative performance across adulthood, with evidence suggesting that several creative indices peak before midlife and may decline in later adulthood (Fusi et al., 2021; Gajda, 2016). Sex has also been examined in relation to both general creative potential and malevolent creativity, although findings remain heterogeneous across domains and measures (Abdulla Alabbasi et al., 2022; Lee & Dow, 2011; Taylor et al., 2024). Finally, education may influence creativity by shaping domain-relevant knowledge, cognitive resources, cultural exposure, and opportunities for creative expression (Amabile, 1983, 1996; Gajda et al., 2017). Based on this rationale, the study hypothesised that relativism would account for the indirect associations between openness to experience and both positive and negative creativity, above and beyond the effects of age, sex, and education (Figure 1).

## 2. Materials and Methods

### 2.1. Participants and Procedure

A convenience sample of 203 psychology students enrolled in bachelor’s and master’s degree programmes was recruited to participate in the study.

The minimum sample size was detected using G*Power 3.1 (Faul et al., 2009). The criteria were number of predictors = 5 (i.e., openness to experience, relativism, age, sex, and education); a medium effect size (f^2^ = 0.15), α = 0.05, and power = 0.95. The minimum required sample size was 138 participants. Participants were eligible if they were at least 18 years old, while exclusion criteria were self-reported history of neurological or psychiatric disorders, substance-related and behavioural addictions.

Outliers were screened and removed based on a ±4.0 z-score criterion (Giancola et al., 2024b; Mertler et al., 2021). This procedure resulted in a final sample of 200 participants (M_age_ = 21.79 years; SD_age_ = 2.64, range: 18–34 years; 146 females). Education was coded as the number of years of formal education completed. In the Italian educational context, this value mainly reflected participants’ progression from upper secondary education (13 years) into bachelor’s (16 years) and master’s (18 years) degree programmes in psychology. The mean level of education was 13.92 years (SD = 1.69). According to self-report socio-demographic information, none of the participants reported a history of neurological or psychiatric disorders, nor substance-related or behavioural addictions.

All participants completed the experimental session individually in a quiet laboratory setting after providing written informed consent. First, they completed a socio-demographic questionnaire, followed by a set of self-report measures, including the Openness to Experience subscale of the HEXACO Personality Inventory–Revised (HEXACO-PI-R) and the Relativism subscale of the Ethics Position Questionnaire (EPQ-5). The order of the self-report measures was counterbalanced across participants to minimise potential order effects. After completing these questionnaires, participants were administered the Cartoon Caption Task. The entire session lasted approximately 60 min. Participation was voluntary, and no financial compensation was provided. The study was conducted in accordance with the Declaration of Helsinki and was reviewed and approved by the Institutional Review Board of the University of L’Aquila (protocol number 39870).

### 2.2. Measures

A brief socio-demographic questionnaire was used to evaluate age, sex, and education.

Openness to experience was assessed using the 10-item subscale from the 60-item HEXACO Personality Inventory–Revised (HEXACO-PI-R; Ashton & Lee, 2009). Items (e.g., I would enjoy creating a work of art, such as a novel, a song, or a painting) were rated on a 5-point Likert-type scale ranging from 1 (completely disagree) to 5 (completely agree). In the present study, the internal consistency of the subscale was acceptable (Cronbach’s α = 0.74). A confirmatory factor analysis was conducted to examine the expected one-factor structure of the subscale. The model showed limited fit to the data, χ^2^(35) = 126.84, *p* < 0.001, CFI = 0.76, TLI = 0.69, RMSEA = 0.12, SRMR = 0.09, with standardised factor loadings ranging from 0.06 to 0.74.

Relativism was measured using the Relativism subscale of the Ethics Position Questionnaire–5 (EPQ-5; Alfieri et al., 2022; O’Boyle & Forsyth, 2021). The subscale includes five items (e.g., What is ethical varies from one situation and society to another), each rated on a 5-point Likert-type scale ranging from 1 (strongly disagree) to 5 (strongly agree). All items were positively worded. In the present study, the internal consistency of the subscale was satisfactory (Cronbach’s α = 0.75). A confirmatory factor analysis was conducted to examine the expected one-factor structure of the scale. The model showed acceptable CFI and SRMR values, although TLI and RMSEA were less satisfactory, χ^2^(5) = 19.38, *p* = 0.002, CFI = 0.94, TLI = 0.88, RMSEA = 0.121, SRMR = 0.06. Standardised factor loadings were positive and ranged from 0.47 to 0.75.

A modified version of the Cartoon Caption Task (Heintz, 2023; Nusbaum et al., 2017) was used to assess positive and negative creativity. In this task, participants viewed five New Yorker-style cartoons showing everyday situations with an incongruous or absurd element. For each cartoon, they were asked to write one caption within 2 min. The task was designed to capture participants’ ability to generate a brief, original, and contextually meaningful response to an ambiguous visual scene.

The five cartoons depicted: (1) an astronaut on the moon speaking on a cell phone; (2) Batman and Superman sitting on a sofa with a psychotherapist; (3) a pirate speaking to his crew while pointing to three boxes of rockets on board; (4) a female detective and a policeman observing a human outline on the floor; and (5) a woman showing unpredictable or irrational behaviour while having breakfast with her surprised husband. Each caption was evaluated for positive and negative creativity. Positive creativity referred to captions that were original, clever, humorous, or contextually appropriate without implying harm. Negative creativity referred to captions that were original but included hostile, aggressive, disturbing, harmful, or socially inappropriate content, even when harm was not explicitly intended. Therefore, the same task allowed the assessment of both constructive and negative creative responses to the same visual stimuli.

Two trained judges (Mage = 20.50; SDage = 0.71; 2 males) independently evaluated all captions. Although previous research has recommended using at least three judges, two well-trained raters can provide reliable creativity scores when training procedures and evaluation criteria are clearly defined (B. T. Christensen & Ball, 2016; Silvia et al., 2008). The judges were undergraduate psychology students recruited from the Department of Biotechnological and Applied Clinical Sciences, University of L’Aquila, through an in-class advertisement. The first two eligible volunteers were selected according to two criteria: (1) completion of a developmental psychology course and (2) a strong interest in creativity research.

As in previous research (Giancola et al., 2021a), judges completed a structured 20 h training programme to ensure standardised evaluation. The training covered: (1) the distinction between divergent thinking, convergent thinking, and creative production; (2) the three creativity criteria used to score the captions: remoteness, cleverness, and uncommonness (Silvia et al., 2008); (3) the distinction between negative creativity, defined as the production of ideas with potentially harmful or socially inappropriate consequences, and malevolent creativity, defined as the intentional use of creativity to harm others for personal gain; and (4) guided scoring practice using sample captions from the Cartoon Caption Task.

Before evaluation, all captions were anonymised. Judges were blind to participants’ socio-demographic information, questionnaire scores, and study hypotheses. Captions were evaluated independently by each judge, and no discussion between judges was allowed during the scoring phase. Each caption was rated separately for positive and negative creativity using the criteria of remoteness, cleverness, and uncommonness on a 5-point Likert-type scale ranging from 1 (not at all creative) to 5 (extremely creative). Captions were presented in a random order and were evaluated independently of the other captions produced by the same participant. All 200 participants completed the Cartoon Caption Task, resulting in 1000 captions. Inter-rater reliability was high for both positive creativity (range = 0.73 to 0.89) and negative creativity (range = 0.70 to 0.90), indicating adequate agreement between judges. Illustrative examples of high-scoring positive and negative captions are provided in the Supplementary Materials.

All instruments used in the current research are fully reported in the Supplementary Materials.

### 2.3. Statistical Analysis

All statistical analyses were conducted using IBM SPSS Statistics (Version 28; IBM Corp., 2019). Preliminarily, descriptive statistics were computed to provide an initial overview of the data and to examine the distributional properties of the study variables. To evaluate the potential impact of common method bias (CMB), Harman’s single-factor test (Harman, 1967; Podsakoff et al., 2003; Podsakoff et al., 2024) was performed by entering all variables into an exploratory factor analysis. Bivariate correlations were also computed to primarily examine the relationships among openness to experience, relativism, positive and negative creativity, and socio-demographics.

To test the mediating role of relativism in the relationship between openness to experience and both positive and negative creativity, mediation analyses were conducted using the PROCESS macro for SPSS (Version 3.5; Hayes, 2017). This choice was made because positive and negative creativity were conceptualised as theoretically distinct outcomes, differing in their social and ethical valence. In addition, the PROCESS model used in the present study estimates mediation effects for each dependent variable separately. Therefore, a single model would not have allowed the indirect effect to be tested separately for each creativity domain. In Model 1, positive creativity was the dependent variable, whereas in Model 2, negative creativity was the dependent variable. Age, sex, and education were included as covariates in both models. Indirect effects were estimated using a bias-corrected bootstrap procedure with 5000 resamples and 95% confidence intervals (CIs). The bootstrapping method treats the sample as a pseudo-population that mirrors the larger population from which it originated, eliminating the need to assume the shape of the sampling distribution in inferential tests (Preacher & Hayes, 2008). This method is a non-parametric approach that enables the testing of moderation and mediation (Giancola et al., 2023a) effects across small to large-sized samples, bypassing the issue of non-normality (Bollen & Stine, 1990).

## 3. Results

### 3.1. Preliminary Analysis

Harman’s single-factor test revealed that the variance explained by the unrotated factor solution accounted for 26.08% of the total variance, suggesting no CMB problems (test critical threshold ≥ 50%). As shown in Table 1, openness to experience was positively associated with relativism (*r* = 0.15, *p* < 0.05) and positive creativity (*r* = 0.16, *p* < 0.05). Relativism showed a positive correlation with negative creativity (*r* = 0.17, *p* < 0.05). Positive creativity was positively correlated with negative creativity (*r* = 0.32, *p* < 0.01), suggesting that the two dimensions share some common variance while remaining distinct constructs. Positive creativity was also positively correlated with age (*r* = 0.16, *p* < 0.01) and education (*r* = 0.21, *p* < 0.01).

### 3.2. Exploratory Stimulus-Level Analysis of Creative Performance

Additional exploratory analyses were conducted to examine whether creative performance was consistent across the five cartoons and whether creativity scores differed between stimuli. For positive creativity, internal consistency across cartoons was modest (Cronbach’s α = 0.57). Intercorrelations among cartoon-specific positive creativity scores were heterogeneous, ranging from very weak to moderate associations. Significant correlations emerged between the astronaut cartoon and the Batman–Superman cartoon (*r* = 0.58, *p* < 0.001), between the astronaut cartoon and the detective cartoon (*r* = 0.54, *p* < 0.001), between the Batman–Superman cartoon and the detective cartoon (*r* = 0.51, *p* < 0.001), and between the detective cartoon and the breakfast cartoon (*r* = 0.19, *p* = 0.008). However, several other correlations were weak and non-significant, suggesting only partial consistency of positive creative performance across stimuli. A Friedman test showed that positive creativity scores did not differ significantly across the five cartoons (χ^2^(4) = 5.49, *p* = 0.240, Kendall’s W = 0.007).

For negative creativity, internal consistency across cartoons was acceptable (Cronbach’s α = 0.73). Intercorrelations among cartoon-specific negative creativity scores were all positive and significant, ranging from *r* = 0.19 to *r* = 0.84, suggesting greater consistency of negative creative performance across stimuli. A Friedman test showed that negative creativity scores differed significantly across the five cartoons (χ^2^(4) = 145.00, *p* < 0.001, Kendall’s W = 0.181). Mean ranks indicated that the pirate cartoon elicited the highest negative creativity scores, followed by the Batman–Superman cartoon, whereas the astronaut, detective, and breakfast cartoons showed lower and relatively similar ranks. Bonferroni-adjusted pairwise comparisons indicated that the Batman–Superman cartoon elicited higher negative creativity than the astronaut cartoon (*p* adj = 0.013), the detective cartoon (*p* adj = 0.017), and the breakfast cartoon (*p* adj = 0.011). The pirate cartoon also elicited higher negative creativity than the astronaut cartoon (*p* adj < 0.001), the detective cartoon (*p* adj < 0.001), and the breakfast cartoon (*p* adj < 0.001). No significant difference emerged between the Batman–Superman and pirate cartoons or among the astronaut, detective, and breakfast cartoons.

### 3.3. Mediation Analysis

Two separate mediation models were tested to examine whether relativism mediated the association between openness to experience and creativity. In Model 1, positive creativity was entered as the dependent variable, whereas in Model 2, negative creativity was entered as the dependent variable (see Table 2). In both models, openness to experience was specified as the independent variable and relativism as the mediator. Age, sex, and education were included as covariates.

In the first regression equation, openness to experience significantly predicted relativism (B = 0.18, SE = 0.09, β = 0.15, t = 2.16, *p* = 0.032, 95% CI [0.02, 0.35]). Among the covariates, sex was significantly associated with relativism (B = 0.28, SE = 0.12, β = 0.16, t = 2.33, *p* = 0.021, 95% CI [0.04, 0.52]), whereas age (B = −0.03, SE = 0.03, β = −0.10, t = −1.10, *p* = 0.274, 95% CI [−0.08, 0.02]), and education (B = 0.04, SE = 0.04, β = 0.09, t = 1.06, *p* = 0.290, 95% CI [−0.04, 0.12]), were not significant. This model explained 5.7% of the variance in relativism, R^2^ = 0.06, F(4, 195) = 2.92, *p* = 0.022.

In Model 1, relativism did not significantly predict positive creativity (B = −0.05, SE = 0.05, β = −0.07, t = −1.01, *p* = 0.316, 95% CI [−0.16, 0.05]). Openness to experience had a significant direct effect on positive creativity (B = 0.16, SE = 0.06, β = 0.18, t = 2.59, *p* = 0.010, 95% CI [0.04, 0.29]). Among the covariates, sex was significantly associated with positive creativity (B = 0.20, SE = 0.09, β = 0.16, t = 2.22, *p* = 0.027, 95% CI [0.02, 0.38]). Age (B = 0.001, SE = 0.02, β = 0.01, t = 0.07, *p* = 0.942, 95% CI [−0.04, 0.04]), and education (B = 0.05, SE = 0.03, β = 0.14, t = 1.63, *p* = 0.104, 95% CI [−0.01, 0.11]), were not significant predictors. The indirect effect of openness to experience on positive creativity through relativism was not significant (B = −0.01, BootSE = 0.01, β = −0.01, 95% BootCI [−0.04, 0.01]). The total effect of openness to experience on positive creativity was significant (B = 0.15, SE = 0.06, β = 0.17, t = 2.47, *p* = 0.014, 95% CI [0.03, 0.28]). The full model explained 8.0% of the variance in positive creativity, R^2^ = 0.08, F(5, 194) = 3.37, *p* = 0.006.

In Model 2, relativism significantly predicted negative creativity (B = 0.11, SE = 0.04, β = 0.22, t = 3.05, *p* = 0.003, 95% CI [0.04, 0.18]). The direct effect of openness to experience on negative creativity was not significant (B = 0.06, SE = 0.04, β = 0.10, t = 1.37, *p* = 0.173, 95% CI [−0.03, 0.14]). None of the covariates significantly predicted negative creativity: age (B = −0.003, SE = 0.01, β = −0.02, t = −0.20, *p* = 0.843, 95% CI [−0.03, 0.02]), sex (B = 0.06, SE = 0.06, β = 0.07, t = 1.03, *p* = 0.305, 95% CI [−0.06, 0.18]), and education (B = 0.01, SE = 0.02, β = 0.04, t = 0.43, *p* = 0.665, 95% CI [−0.03, 0.05]). The indirect effect of openness to experience on negative creativity through relativism was significant (B = 0.02, BootSE = 0.01, β = 0.03, 95% BootCI [0.001, 0.05]). The total effect of openness to experience on negative creativity was not significant (B = 0.08, SE = 0.04, β = 0.13, t = 1.82, *p* = 0.071, 95% CI [−0.01, 0.17]). The full model explained 7.5% of the variance in negative creativity, R^2^ = 0.08, F(5, 194) = 3.15, *p* = 0.009.

Overall, the results indicated that relativism mediated the association between openness to experience and negative creativity, but not the association between openness to experience and positive creativity. These effects were observed while controlling for age, sex, and education.

To further examine the robustness and specificity of the Model 2, a robustness check was conducted by performing two alternative mediation models. In the first alternative model, relativism was entered as the independent variable, openness to experience as the mediator, and negative creativity as the dependent variable. The indirect effect of relativism on negative creativity through openness to experience was not significant (B = 0.01, BootSE = 0.01, β = 0.01, 95% BootCI [−0.003, 0.02]). In the second alternative model, openness to experience was entered as the independent variable, negative creativity as the mediator, and relativism as the dependent variable. The indirect effect of openness to experience on relativism through negative creativity was not significant (B = 0.03, BootSE = 0.02, β = 0.03, 95% BootCI [−0.002, 0.08]). Overall, these robustness checks indicated that the indirect association was observed only in the model, in which relativism accounted for the association between openness to experience and negative creativity.

## 4. Discussion

The present study examined whether relativism accounted for the association between openness to experience and two forms of creativity, namely positive and negative creativity.

The findings partially supported the research hypothesis as indirect effect was observed exclusively for negative creativity, whereas no indirect effects emerged for positive creativity. Despite the limited factorial validity of the scales, these patterns suggest that the main features of openness to experience, such as curiosity, flexibility, and imagination are associated with negative creativity, when contextual-based moral evaluations, underpinned by relativism, are involved. Indeed, individuals high in openness to experience may be more inclined to adopt a context-dependent moral framework, in which situational factors, anticipated consequences, and social expectations are prioritised over universal ethical rules when making judgements (D. Forsyth, 2020; O’Boyle & Forsyth, 2021). Within this context, individuals may evaluate actions and outcomes in relation to personal interests, benefits, or contextual demands without necessarily compromising their broader thinking style (Costa & McCrae, 1992). This interpretation aligns with evidence in the field of education, stressing that students’ academic behaviour during ethically ambiguous situations is shaped by the interaction between personality-related features, motivation, and contextual demands (Eshet, 2024).

The absence of the indirect association between openness to experience and positive creativity through relativism and the significance of the direct association between openness to experience and positive creativity suggests a different pattern for the bright side of creative production. This finding indicates that positive creativity may be more closely related to the core features of openness to experience, such as imagination, intellectual curiosity, aesthetic sensitivity, and cognitive exploration (Bierly et al., 2009; Cropley et al., 2008; Cropley, 2010). Generating humorous, clever, original, or contextually appropriate captions may rely more on dispositional open-mindedness than on relativistic moral reasoning. In contrast, negative creativity may involve an additional ethical component, as negative creative responses require originality and a willingness to entertain ideas that depart from socially desirable or morally conventional expectations.

From a theoretical standpoint, the findings contribute to a more nuanced understanding of the psychological correlates associated with different forms of creativity. The indirect association between openness to experience and negative creativity via relativism is consistent with the Investment Theory of Creativity (ITC; Giancola et al., 2023b; Sternberg & Lubart, 1991, 1993). The ITC posits that creativity results from the blend of multiple cognitive, personality, and environmental factors (Sternberg, 2009, 2020). Additionally, the findings align with the AMORAL Model (Kapoor & Kaufman, 2022), which conceptualises morally ambiguous creativity as emerging from the interplay of dispositional traits, cognitive flexibility, and sociocultural factors. The present results further suggest that ethical orientation, specifically relativism, may represent an additional psychological factor of creative expression. Overall, these insights highlight the value of integrative frameworks that explicitly incorporate moral cognition in models of creativity.

From an educational perspective, the results suggest the importance of promoting ethical awareness along with creative thinking. Since ethically ambiguous creative ideation may be associated with both dispositional openness to experience and relativism, creativity training programmes should not focus exclusively on originality and divergent thinking. They should also encourage reflection on the social, interpersonal, and moral consequences of creative ideas. Integrating moral reasoning tasks, ethical dilemmas, discussions of academic integrity, and socio-moral reflection into creativity curricula may help students evaluate whether an idea is original, responsible, constructive, and socially appropriate. This approach would also acknowledge the role of education in shaping creativity by providing students with domain-relevant knowledge, ethical awareness, cultural exposure, and opportunities for responsible creative expression (Amabile, 1983, 1996; Gajda et al., 2017).

Despite these implications, several limitations and future research directions should be considered when interpreting these findings. First, the cross-sectional design precludes causal inference. Future longitudinal research is needed to clarify the directionality of the relationships among openness to experience, relativism, and both positive and negative creativity. Second, the sample was predominantly female and consisted of undergraduate and master’s psychology students. Although this sex distribution is common in psychology and other social science programmes (Charles & Bradley, 2009), it may limit the generalisability of the findings. Future studies should replicate these findings in larger, more heterogeneous samples, including participants from diverse academic fields and with a more balanced sex distribution. Third, the observed effect sizes in the mediation models were relatively small, warranting caution in interpreting and generalising the findings. The explained variance in both mediation models remains relatively modest (approximately 8%), suggesting that additional mediating or moderating factors should be considered in future studies. Furthermore, the present study examined the role of relativism in relation to a single personality trait (i.e., openness to experience). Although openness to experience represents a relevant dispositional factor of creativity (Tan et al., 2019), future research should adopt a broader individual-differences perspective to clarify whether relativism operates similarly across other psychological characteristics. In particular, cognitive styles may represent a promising avenue for further investigation, given their influence on individual performance across different domains (Aggarwal et al., 2023; Bocchi et al., 2018; Giancola et al., 2021b; Peterson & Meissel, 2015; Phillips et al., 2016), including creative thinking and creative production (Miller, 2007; Giancola et al., 2023c; Ho & Kozhevnikov, 2023). Fourth, the confirmatory factor analysis of the openness to experience subscale and relativism scale showed limited model fit, suggesting that the factorial validity of this measure in the present sample should be interpreted with caution. Fifth, the distribution of negative creativity scores was skewed, with higher ratings (e.g., 4 and 5 on the Likert scale) notably absent. This may be attributed to the general nature of the task instructions, which did not explicitly encourage participants to engage in or express negative creativity. As a result, responses may have been biased toward more socially acceptable or prosocial expressions of creativity. This aligns with prior research by Kapoor and Khan (2016), who found that participants generated fewer than 2% of negative original responses even when using the alternative uses task. This limitation constrains how the findings can be generalised to more deliberate or extreme manifestations of negative creativity. Sixth, the Cartoon Caption Task measured hypothetical caption generation rather than real-world harmful creative behaviour. Therefore, the findings cannot be directly extended to actual harmful behaviour, deception, manipulation, aggression, or real-world ethical misconduct. The task is useful for capturing negative creative ideation under controlled conditions, but it does not assess whether participants would enact harmful creative behaviours outside the laboratory. Related to this issue, future research should compare neutral instructions with explicit negative-creativity instructions to determine whether relativism is more strongly associated with negative creativity when participants are invited to generate unconventional, transgressive, or socially inappropriate ideas.

## 5. Conclusions

The present study examined the ethical dimension of creativity by exploring how openness to experience and relativism are associated with positive and negative creativity. The findings showed that relativism accounted only for the association between openness to experience and negative creativity. This suggests that negative creative ideation may be linked to the combination of dispositional openness and context-dependent ethical beliefs. By contrast, positive creativity appeared to be more directly associated with openness to experience.

Overall, these findings contribute to the literature on individual differences in creativity by suggesting that ethical orientation may help explain why some forms of creative ideation take on a more negative or morally ambiguous valence.

## Figures and Tables

**Table 1:** Descriptive statistics and correlation among all variables measured.

	M	SD	1.	2.	3.	4.	5.	6.	7.
Openness to experience	3.48	0.64	1						
2. Relativism	3.38	0.77	0.15 *	1					
3. Positive creativity	2.16	0.57	0.16 *	−0.05	1				
4. Negative creativity	1.22	0.39	0.09	0.17 *	0.32 **	1			
5. Age	21.79	2.64	0.15 *	−0.06	0.16 **	0.02	1		
6. Sex			−0.05	0.16 *	0.13	0.10	−0.08	1	
7. Education (years)	13.92	1.69	0.20 **	0.01	0.21 **	0.09	0.60 **	−0.06	1

Note. N = 200. Sex (0 = F; 1 = M) was dummy coded. Only for sex the point-biserial correlation was used. * *p* < 0.05 (two tailed). ** *p* < 0.01 (two tailed).

**Table 2:** Summary of the mediation analyses.

	B	SE	β	t	*p*	95% CI
Model 1: Positive creativity						
Path a (Openness to experience → Relativism)	0.18	0.09	0.15	2.16	0.032	[0.02, 0.35]
Path b (Relativism → Positive creativity)	−0.05	0.05	−0.07	−1.01	0.316	[−0.16, 0.05]
Indirect effect (*a* × *b*)	−0.01	0.01	−0.01			[−0.04, 0.01]
Direct effect (*c’*)	0.16	0.06	0.18	2.59	0.010	[0.04, 0.29]
Total effect (*c*)	0.15	0.06	0.17	2.47	0.014	[0.03, 0.28]
R^2^ = 0.08 (F(5, 194) = 3.37, *p* = 0.006)						
Model 2: Negative creativity						
Path a (Openness to experience → Relativism)	0.18	0.09	0.15	2.16	0.032	[0.02, 0.35]
Path b (Relativism → Negative creativity)	0.11	0.04	0.22	3.05	0.003	[0.04, 0.18]
Indirect effect (*a* × *b*)	0.02	0.01	0.03			[0.001, 0.05]
Direct effect (*c’*)	0.06	0.04	0.10	1.37	0.173	[−0.03, 0.14]
Total effect (*c*)	0.08	0.04	0.13	1.82	0.071	[−0.01, 0.17]
R^2^ = 0.075 (F(5, 194) = 3.15, *p* = 0.009)						

Note. B = unstandardised coefficient; β = standardised coefficient; SE = standard error; BootSE = bootstrap standard error; CI = confidence interval; BootCI = bootstrap confidence interval. Standardised coefficients for indirect effects refer to completely standardised indirect effects. Path “*a*” represents the association between openness to experience and relativism. Path “*b*” represents the association between relativism and the respective creativity outcome. The indirect effect was estimated using 5000 bootstrap samples. Age, sex, and education were included as covariates in both models.

## Data Availability

The raw data supporting the conclusions of this article will be made available by the authors on request.

## Supplementary Materials

The following supporting information can be downloaded at: https://www.mdpi.com/article/10.3390/bs16071165/s1.
