# Supplementary material for: Ethical Relativism Mediates the Association Between Openness to Experience and Negative Creativity
Source: Behav Sci (Basel). 2026 Jul 10;16(7):1165. doi: 10.3390/bs16071165 (PMC13406019; doi:10.3390/bs16071165)
Supplement: Supplementary file 1 [file behavsci-16-01165-s001.zip › behavsci-4344043-supplementary.pdf]

Supplementary Materials

HEXACO-PI-R (Openness subscale)

Instructions: On the following pages you will find a series of statements about you. Please read each statement and decide how much you agree or disagree with that statement. Then write your response in the space next to the statement using the following scale:

1 = strongly disagree
2 = disagree
3 = neutral (neither agree nor disagree)
4 = agree
5 = strongly agree
Please answer every statement, even if you are not completely sure of your response.

Items:

1. I would be quite bored by a visit to an art gallery.
2. I'm interested in learning about the history and politics of other countries.
3. I would enjoy creating a work of art, such as a novel, a song, or a painting.
4. I think that paying attention to radical ideas is a waste of time.
5. If I had the opportunity, I would like to attend a classical music concert.
6. I’ve never really enjoyed looking through an encyclopedia.
7. People have often told me that I have a good imagination.
8. I like people who have unconventional views.
9. I don’t think of myself as the artistic or creative type.
10. I find it boring to discuss philosophy.

The Short Ethics Position Questionnaire (EPQ-5)

Instructions: You will find a series of general statements listed below. Each represents a commonly held opinion and there are no right or wrong answers. You will probably disagree with some items and agree with others. We are interested in the extent to which you agree or disagree with such matters of opinion.
Please read each statement carefully. Then indicate the extent to which you agree or disagree where:
1 = Strongly disagree
2 = Disagree
3 = Neutral
4 = Agree
5 = Strongly Agree

Items:

1. A person should make certain that their actions never intentionally harm another even to a small degree.
2. The existence of potential harm to others is always wrong, irrespective of the benefits to be gained.
3. One should never psychologically or physically harm another person.
4. One should not perform an action which might in any way threaten the dignity and welfare of another individual.
5. If an action could harm an innocent other, then it should not be done.
6. What is ethical varies from one situation and society to another.
7. Moral standards should be seen as being individualistic; what one person considers to be moral may be judged to be immoral by another person.
8. Questions of what is ethical for everyone can never be resolved since what is moral or immoral is up to the individual.
9. Moral standards are simply personal rules that indicate how a person should behave, and are not to be applied in making judgments of others.
10. Ethical considerations in interpersonal relations are so complex that individuals should be allowed to formulate their own individual codes.

Cartoon Caption Task

Instructions: For this task, write something that is as funny as possible, i.e. strange, silly, crude, bizarre, ironic, perverse, the important thing is that it is funny. For each of the 5 images below, create a title that is funny.

Brief textual description of the stimuli used in the Cartoon Caption Task

Stimulus 1: An astronaut sitting on the moon while speaking on a mobile phone.

Stimulus 2: Batman and Superman sitting together on a sofa during a psychotherapy session.

Stimulus 3: A pirate addressing his crew on a boat while showing several boxes of rockets.

Stimulus 4: A female detective and a police officer examining a human-shaped outline marked on the floor.

Stimulus 5: A surprised husband and an agitated wife having breakfast together at home.

Illustrative examples of high-scoring positive and negative captions in the Cartoon Caption Task

An example of positive creativity for the cartoon depicting an astronaut on the moon speaking on a cell phone is: “Finally, no roaming charges!”. This caption can be considered highly creative. First, it demonstrates uncommonness, as a few people would spontaneously associate space travel with the technicalities of mobile phone billing. Second, it reflects remoteness, by bridging two conceptually distant domains – space exploration and everyday concerns about cell phone costs. Finally, it shows cleverness, as the humour arises from ironic incongruity: the astronaut is in an extraordinary, historic context, yet trivialises it by focusing on a mundane and comically inappropriate detail.

An example of negative creativity for the cartoon depicting an astronaut on the moon speaking on a cell phone is: “Do not worry, the Earth already exploded – you’re the last one left”. This caption can be rated highly creative first in terms of uncommonness because instead of associating the astronaut using a cell phone with poor signal, loneliness, and futuristic technology, it associates the astronaut with the catastrophic destruction of Earth. Second, the caption reflects remoteness because it bridges the mundane act of making a reassuring phone call, and a catastrophic scenario. Finally, it shows cleverness because the speaker does not demonstrate any worry for the destruction of humanity, as the event was casual in nature. The contrast between reassurance and catastrophe makes the caption memorable.
